# Health System Resilience: What Are We Talking About? A Scoping Review Mapping Characteristics and Keywords

**DOI:** 10.15171/ijhpm.2019.71

**Published:** 2019-09-17

**Authors:** My Fridell, Sanna Edwin, Johan von Schreeb, Dell D. Saulnier

**Affiliations:** Department of Public Health Sciences, Karolinska Institutet, Stockholm, Sweden.

**Keywords:** Health System Resilience, Scoping Review, Shocks, Health System

## Abstract

**Background:** Health systems are based on 6 functions that need to work together at all times to effectively deliver safe and quality health services. These functions are vulnerable to shocks and changes; if a health system is unable to withstand the pressure from a shock, it may cease to function or collapse. The concept of resilience has been introduced with the goal of strengthening health systems to avoid disruption or collapse. The concept is new within health systems research, and no common description exists to describe its meaning. The aim of this study is to summarize and characterize the existing descriptions of health system resilience to improve understanding of the concept.

**Methods and Analysis:** A scoping review was undertaken to identify the descriptions and characteristics of health system resilience. Four databases and gray literature were searched using the keywords "health system" and "resilience" for published documents that included descriptions, frameworks or characteristics of health system resilience. Additional documents were identified from reference lists. Four expert consultations were conducted to gain a broader perspective. Descriptions were analysed by studying the frequency of key terms and were characterized by using the World Health Organization (WHO) health system framework. The scoping review identified eleven sources with descriptions and 24 sources that presented characteristics of health system resilience. Frequently used terms that were identified in the literature were shock, adapt, maintain, absorb and respond. Change and learning were also identified when combining the findings from the descriptions, characteristics and expert consultations. Leadership and governance were recognized as the most important building block for creating health system resilience.

**Discussion:** No single description of health system resilience was used consistently. A variation was observed on how resilience is described and to what depth it was explained in the existing literature. The descriptions of health system resilience primarily focus on major shocks. Adjustments to long-term changes and the element of learning should be considered for a better understating of health system resilience.

## Background

A health system “consists of all organizations, people and actions whose primary intentis to promote, restore or maintain health.”^1^ A World Health Organization (WHO) framework summarizes the functions of a health system as 6 interconnected building blocks that are essential to health system functioning.^1^ The building blocks must work together to respond to changing health needs so that the system can reach the health system goal of improving health.^1,2^

Recent challenges, such as shifting burden of disease, antimicrobial resistance, financial crises and an increasing frequency of extreme weather events, have put pressure on the functions of health systems.^3^ These challenges range from national or local disruptions to pandemics with substantial global impact, such as the West Africa Ebola outbreak from 2014-2016, which resulted not only in a substantial loss of life but also in severe impediments to the functioning of the health systems in the affected countries.^4^ Similar patterns of a health system’s inability to cope have also been seen in countries affected by the Zika virus.^5,6^ Although these diseases mainly affected low- and middle-income countries, other difficulties have affected high-income countries, such as the 2008-2009 financial crisis that forced multiple health systems in Europe to reorganize for better efficiency and to make cutbacks in health budgets.^7,8^ Further challenges are slowly emerging worldwide that are causing progressive stress to systems, for instance the continued threat of antibiotic resistance that is predicted to become one of the largest challenges to health systems in the future.^9^

The term *resilience* has been part of the lexicon of multiple scientific disciplines; psychology, disasters, engineering, and economics, for instance, all use frameworks of resilience.^10-12^ One of the first disciplinary definitions of resilience came from the field of ecology in 1973, where it was defined by Holling as a system’s “ability to absorb change and disturbance and still maintain the same relationships between populations or state variables.”^13^ From this definition and definitions from other disciplines, the main concepts of resilience emerged – the capacity of an individual, population or system to absorb a shock, while still retaining the fundamental functions or characteristics of the original state.^14,15^ However, this view of resilience has been critiqued for seeing a system as a strictly linear process, with the underlying assumption that the original state of the system is the optimal state, to which a system should return after being shocked into departing from it.^16,17^ In this scenario, a system can only absorb shocks, and remains vulnerable. A more dynamic interpretation of resilience incorporates adaptive and transformative capabilities that allow a system to adjust or change its own characteristics or actions to soften future shocks while still retain its basic structure, or even fundamentally change its structure to eliminate risks altogether, if its current state becomes unsustainable.^18^

While the term has been in use for many years, it was when the negative effects of the West Africa Ebola outbreak on local health systems was documented that the term gained popularity as a concept. Health system resilience was swiftly taken up within global health as a way to strengthen health systems,^4^ focusing primarily on acute shocks to the health system, such as the Ebola outbreak and natural disasters.^19^ This focus has broadened since to include health systems facing chronic stresses that continuously challenge the performance of the system or its ability to adapt.^20^ However, specific suggestions on how health systems can become resilient to either acute shocks or chronic stress remain comparatively vague.^11^

There is no common description of health system resilience at this stage.^19^ In order to build resilient health systems, a better understanding of what it means and should contain is needed.^21^ Clarifying the meaning of health system resilience could help establish a shared understanding of the concept among researchers and policy-makers.^10^ The purpose of this study is therefore to summarize and characterize the existing descriptions of health system resilience.

## Methods

This study combines a scoping review of peer-reviewed and gray literature with expert consultations.^22^ A scoping review was chosen due to the broad nature of the concept and to capture the variety in the published information on health systems resilience. Expert consultations were used to supplement the literature and to gain practical perspectives of health systems resilience.^22,23^

The literature search for peer-reviewed articles was conducted in January 2018 and the gray literature search in March 2018. Search strategies for peer-reviewed literature and gray literature were developed. Both used the search keywords of *resilience* and *health systems* (Table 1). The primary inclusion criteria were documents that were published in English and described or identified the characteristics of health system resilience or that presented a health system resilience framework (Table 2).

**Table 1:** Search Terms, by Database

**Source**	**Type of Literature**	**Search Terms**
PubMed	Peer-reviewed	(resilien*[tiab] AND health system*[tiab])
Web of Science	Peer-reviewed	TS = (resilien* NEAR/3 health system*)
Global Health	Peer-reviewed	(health system*) AND (resilien*)
IRIS	Gray literature	Resilient OR resilience AND health system* title contains resilient OR resilience title contains health system
Google	Gray literature	Health system resilience

Abbreviation: IRIS, Institutional Repository for Information Sharing.

**Table 2:** Eligibility Criteria for the Review

**Criteria for Inclusion**
Full text written in English
Articles, reports, books, opinion papers, workshop summaries, briefings, commentaries, or webpages
Gives a written description of health system resilience
Includes a visual representation or discussion of a framework for health system resilience
Identifies or discusses different characteristics, concepts, elements or components of health system resilience
**Criteria for Exclusion**
Abstracts, videos, or news articles

For the peer-reviewed literature, searches were conducted in the PubMed, Web of Science, and Global Health databases. The gray literature search followed the methodology of Godin et al^24^ for a reproducible search. A keyword search was used in the Institutional Repository for Information Sharing (IRIS) database, and a second search was conducted through Google. Organizations that work with health systems or public health were identified from the first 50 hits of a Google search by using the term “health system resilience.” The organization webpages search engines were used to find relevant documents by using the same search string. In the absence of a search engine, the website of the organization was manually searched. After sorting the results by relevance, the first 50 documents from each organization were included for screening.

**Figure 1:**
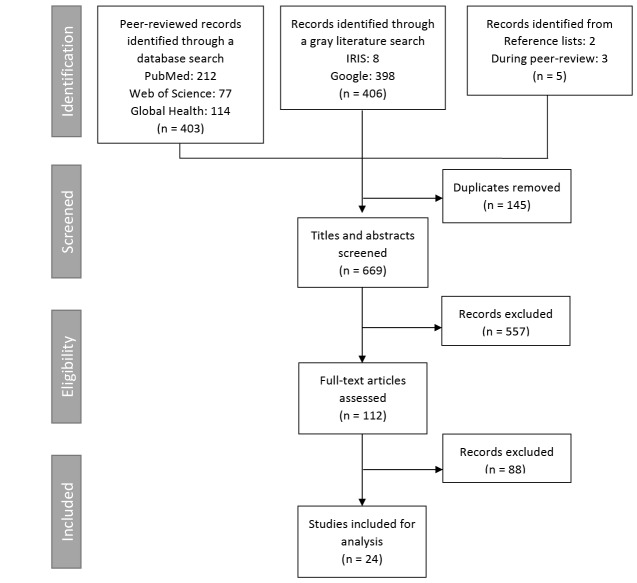
PRISMA Diagram of the Literature Search. Abbreviation: IRIS, Institutional Repository for Information Sharing.

The initial searches produced 811 articles. The first 2 authors independently assessed the documents for inclusion. After screening the titles and abstracts, 112 articles were read in full, and 88 were excluded (Figure 1). Additional references were identified from the reference lists of the included articles, and 3 articles were identified during the peer-review process and included in the analysis. In case of a disagreement on the inclusion of a document, the document was discussed among the authors until an agreement was reached. The data were extracted from the included studies and entered into an Excel spreadsheet by the first 2 authors. No quality assessment of the articles was done, as this is not part of the standard methodology of scoping reviews.^22^

Expert consultations were performed during March and April 2018, with experts who were affiliated with an organization, institute or governmental agency that was identified as relevant to health system and policy. The experts were purposively sampled so that they held a position in the field of health systems and policy and were familiar with the concept of health system resilience. The organizations of the experts were identified through the gray literature search, and no organization had more than one representative. Experts were contacted by email for recruitment and each one agreed to participate. Experts were asked to describe and identify the characteristics of health system resilience before the interview was conducted. Additional questions during the interviews used the preliminary findings from the literature review but were not provided to the experts beforehand to avoid potential bias. The consultations were held online, audio recorded, and transcribed by intelligent verbatim.^25^

The data from the literature search and expert consultations were analysed by the first 2 authors by mapping the constructs within the descriptions and comparing the occurrence of constructs between the different descriptions.^26^ Keywords within each description and the resilience characteristics were identified and charted; synonyms were combined into one category and were named for the most frequently used term within this category. Keywords were included in the analysis if they were mentioned in 3 or more articles and interviews, in order to prioritize the words used most frequently and to prevent seldomly used words from influencing the results. The characteristics of health system resilience from the different publications were identified and mapped into one of the WHO’s 6 building blocks.^1^ The WHO 6 building block framework was chosen for its cohesiveness, as it has been a commonly used framework for health systems resilience research.

## Results

The final analysis included 24 publications that were published between 2013 and 2018. Four experts who were affiliated with WHO, the United Nations Children’s Fund (UNICEF), the European Observatory on Health Systems and Policies, and the Swedish Ministry for Foreign Affairs were consulted.

Eleven of the included articles provided a description of health system resilience (Table 3), 2 articles referenced a description from another paper, 6 articles referred to a description about resilience in general and 5 articles did not provide a description.

**Table 3:** Health System Resilience Descriptions and Keywords Used in the Included Articles

**Abimbola et al, 2018**	Adaptation with robustness: the case for clarity on the use of ‘resilience’ in health systems and global health
*Description* Resilience implies adaptability in a context of robustness	*Context* Governance for resilience *Keywords used* Adapt
**Ammar et al, 2016**	Health system resilience: Lebanon and the Syrian refugee crisis
*Description* The capacity to absorb internal and external shocks and maintain functional health institutions while sustaining achievements	*Context* Refugee crisis in Lebanon
	*Keywords used* Absorb; Shock^*^; Maintain
**Barasa et al, 2017**	From bouncing back, to nurturing emergence: reframing the concept of resilience in health systems strengthening
*Description* Resilience is about (1) everyday resilience, not simply response to sudden shocks, (2) health system software, not only its hardware, and (3) creative adaptation, and transformation, rather than simply bouncing back	*Context* Everyday resilience
	*Keywords used* Response; Shock^*^; Adapt^**^; Transform
**Barasa et al, 2018**	What is resilience and how can it be nurtured? A systematic review of empirical literature on organizational resilience
*Description* Refers to organizational resilience, not specific to health systems	*Context* Organizational resilience
	*Keywords used*
**Blanchet et al, 2017**	Governance and capacity to manage resilience of health systems: towards a new conceptual framework
*Description* Its capacity to adapt, absorb and transform when exposed to a shock such as a pandemic, natural disaster, armed conflict or a financial crisis and still retain the same control over its structure and functions	*Context* Governance for resilience
	*Keywords used* Adapt^**^; Absorb; Transform; Shock; Maintain^***^
**Blanchet, 2013**	Governance of health systems comment on “A network based theory of health systems and cycles of well-being”
*Description* Refers to resilience, not specific to health systems	*Context* Governance for resilience
	*Keywords used*
**Campbell et al, 2015**	Improving the resilience and workforce of health systems for women’s, children’s, and adolescents’ health
*Description* Its capacity to respond, adapt, and strengthen when exposed to a shock, such as a disease outbreak, natural disaster, or conflict	*Context* Women’s, children’s and adolescent health
	*Keywords used* Respond; Adapt^**^; Shock^*^
**European Commission, 2014**	Communication from the commission: On effective, accessible and resilient health systems
*Description* Able to adapt effectively to changing environments and tackling significant challenges with limited resources	*Context* Action plan for resilience
	*Keywords used* Adapt^*^
**Gilson et al, 2017**	Everyday resilience in district health systems: emerging insights from the front lines in Kenya and South Africa
*Description* Refers to resilience, not specific to health systems	*Context* Everyday resilience
	*Keywords used*
**Hanefeld et al, 2018**	Towards an understanding of resilience: responding to health systems shocks
*Description* Able to adapt its functioning to absorb a shock and transform if necessary to recover from disaster	*Context* Understanding health system resilience
	*Keywords used* Adapt^**^; Absorb; Shock^*^; Transform
**Ho et al, 2016**	Applying the resilient health system framework for universal health coverage
*Description* None	*Context* Digital healthcare resilience
	*Keywords used*
**International Forum Gastein , 2013**	Resilient and innovative health systems for Europe
*Description* None	*Context* Health system innovations
	*Keywords used*
**Khan et al, 2017**	A review on the antagonist Ebola: a prophylactic approach
*Description* Refers to resilience, not specific to health systems	*Context* West Africa Ebola outbreak
	*Keywords used*
**Kieny et al, 2015**	Beyond Ebola: a new agenda for resilient health systems
*Description* Can absorb the shock of an emergency such as Ebola and simultaneously continue to provide regular health services and leave other sectors of the country fully functioning	*Context* West Africa Ebola outbreak
	*Keywords used* Shock^*^; Absorb
**Kruk et al, 2015**	What is a resilient health system? Lessons from Ebola
*Description* The capacity of health actors, institutions, and populations to prepare for and effectively respond to crises, maintain core functions when a crises hits, and informed by lessons learned during the crisis, reorganize if conditions require it	*Context* West Africa Ebola outbreak
	*Keywords used* Respond; Shock^*^; Maintain^***^; Learn; Adapt^**^
**M8 Alliance Kyoto, 2015**	Meeting emerging challenges: toward responsive and resilient health systems
*Description* None	*Context* Emerging challenges for health systems
	*Keywords used*
**McKenzie et al, 2015**	Building a resilient health system: lessons from Northern Nigeria
*Description* None	*Context* West Africa Ebola outbreak
	*Keywords used*
**Mfutso-Bengo et al, 2017**	Proposing the LEGS framework to complement the WHO building blocks for strengthening health systems: one needs a LEG to run an ethical, resilient system for implementing health rights
*Description* Refers to resilience, not specific to health systems	*Context* Strengthening health systems
	*Keywords used*
**Olu , 2017**	Resilient health system as conceptual framework for strengthening public health disaster risk management: an African viewpoint
*Description* Reference to Kruk et al, 2015	*Context* Disaster management
	*Keywords used*
**Oxfam, 2015**	Never again: building resilient health systems and learning from the Ebola crisis
*Description* Reference to Kieny et al, 2015	*Context* West Africa Ebola outbreak
	*Keywords used*
**Ozawa et al, 2016**	Exploring pathways for building trust in vaccination and strengthening health system resilience
*Description* Able to withstand major shocks and disruptions to quickly adapt to changing circumstances and to maintain a high utilization and demand over time	*Context* Immunization
	*Keywords used* Shock^*^; Adapt^**^; Withstand; Maintain
**RESYST, 2017**	Using intersectionality to better understand health system resilience
*Description* None	*Context* Everyday resilience
	*Keywords used*
**Thomas et al, 2013**	The framework for assessing health system resilience in an economic crisis: Ireland as a test case
*Description* Refers to resilience, not specific to health systems	*Context* Economic resilience
	*Keywords used*
**WHO, 2015**	Operational framework for building climate resilient health systems
*Description* Can anticipate, respond to, cope with, recover from and adapt to climate-related shocks and stress to provide sustained improvements in population health despite an unstable climate	*Context* Climate resilient health systems
	*Keywords used* Shock^*^; Adapt^**^; Maintain^***^; Respond
**Expert 1**	*Organization:* UNICEF
*Description* The ability to withstand shocks, be they natural or man-made disturbances	*Context* Global health
	*Keywords used* Shock^*^; Learn; Withstand
**Expert 2**	*Organization:* Swedish Ministry of Foreign Affairs
*Description* The ability to manage and cope with threats, challenges and emergencies while maintaining the normal functions and services of a health system; a system can also learn from these experiences and develop and evolve its functionality to become even stronger	*Context* Global health
	*Keywords used* Shock^*^; Maintain^***^; Learn
**Expert 3**	*Organization:* WHO Alliance for Health Policy and Systems Research
*Description* Keywords used to describe resilience	*Context* Global health
	*Keywords used* Shock^*^; Adapt^**^; Respond; Learn; Withstand
**Expert 4**	*Organization:* European Observatory on Health Systems and Policies
*Description* Keywords used to describe resilience	*Context* European health policy
	*Keywords used* Shock^*^; Adapt^**^; Respond; Learn; Withstand

Abbreviation: UNICEF, the United Nations Children’s Fund. *Shock includes the keywords of crises, disturbances and threats; **Adapt includes reorganization; *** Maintain includes the keywords of retain and sustain.

Nine out of 11 descriptions mentioned *shocks*, which refer to a crisis or disruption (Table 4). One source described the meaning of a shock,^19^ while the other 8 sources gave examples of what could cause shocks and how they should be addressed.^4,21,27-32^ Commonly used examples of a shock were pandemics such as Ebola^31,33-35^ or natural disasters.^26,36^ Five recurring terms that referred to the capacities or actions that a system could take to be resilient when exposed to a shock were identified in the descriptions of resilience in the literature, namely, *adapt*, *maintain*, *absorb, respond and transform*. The expert consultations identified 2 additional terms, *learn* and *withstand*, that were not commonly used in the literature.

**Table 4:** Explanation of the Key Terms in Relation to Health System Resilience

**Keyword**	**Times Used**		**Explanation**
	**Articles**	**Experts**	
Shock	9	4	A sudden and often surprising event that causes an additional burden to the health system, most often for a short period of time. Pandemics such as Ebola or natural disasters caused by climate change were the commonly used examples of a shock.
Adapt	9	2	How a system reacts to meet the changing needs of the population and to continue to deliver the best possible care. To be resilient, a system should adapt both during and after sudden shocks and long-term changes.
Maintain	5	1	Maintaining the core functions of the health system when managing a shock. All parts of the health system should maintain the same access to and quality of care to be resilient, although resources might be moved towards the response to a shock.
Absorb	4	0	The capacity of a health system to use additional resources when managing a shock and handling challenges in an efficient way. By better absorbing a shock, the health system is less affected and more resilient.
Respond	4	2	The quick reaction and implementation of effective strategies to address a shock before it overwhelms the system. Quickly responding to a shock can prevent the shock from persisting or expanding, allowing a system to more easily combat problems.
Learn	1	4	A health system must learn from previous experiences of shocks and changes, both within its own system and other systems, nationally and internationally, to increase resilience. Without the element of learning and evaluating previous experiences, there would be limited improvements in the preparation for similar situations and thus a system would not improve its resilience.
Transform	3	0	The ability of a health system to make a complete change to improve for the future, when exposed to a long-term challenge or a shock. To be resilient, a health system should transform the current status, strategies and behaviours that are no longer feasible.
Withstand	1	3	Used to describe how a system must be sufficiently strong to cope with a shock and handle the additional strain and normal functions, which links it to the terms respond and maintain. If the system can withstand the shock or change while still providing routine care to the population, the system can be considered resilient.

**Figure 2:**
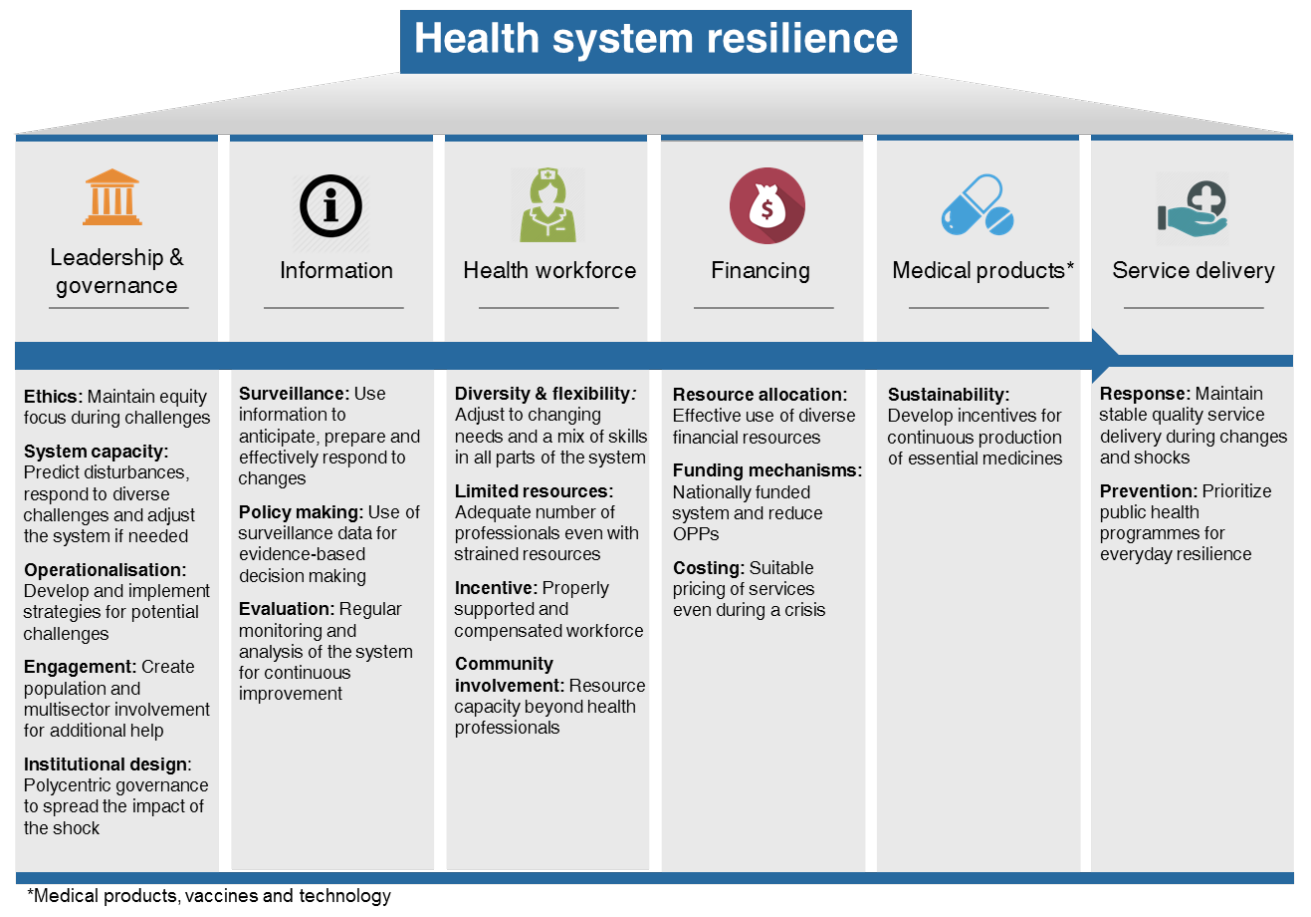
Characteristics of Health System Resilience Within Each of the WHO 6 Building Blocks. Abbreviation: WHO, World Health Organization.

In addition to the keywords used when describing resilience, the publications described health system characteristics that can lead to resilience (Figure 2). However, several characteristics were not concerned with resilience during a shock but with the periods in between a shock.^27,28,32,37-39^ This was described as *everyday resilience*, where the context is not a shock but a chronic challenge that occurs every day over a long period of time and that cannot always be predicted.^20,48-50^

### Financing

Effective financial resource allocation and protection of healthcare funding was identified as important for resilience during shocks, long-term changes and normal strains.^8,27,30,40,41^ Using diverse and stable financial resources was recognized as minimizing the risk of a response being underfunded.^19,30,40^ To be sustainable and able to provide universal health coverage, Hanefeld et al and Kamal-Yanni et al both suggested that a resilient health system should be nationally funded, preferably through taxes.^19,33^ Kamal-Yanni et al additionally suggested that the amount of out-of-pocket payments should be reduced to minimize the inequity in the affordability of health services and to protect from impoverishment due to healthcare costs.^33^ In addition, the importance of selling medical products at stable prices during shocks was highlighted.^40^

### Health Workforce

Five articles discussed the need for a workforce with a mix of skills that can adjust to both long-term changes and shocks.^19,34,35,40,42^ The competence within the health workforce should be sufficiently high to maintain the daily functions of the health system and still provide quality care, even when resources may be scarce.^19,21,40^ The availability of additional workforce resources was raised as a method to improve the speed and effectiveness of the response to a shock while minimizing the negative impact on the system.^21,30,31,33,38^ Communities were also identified as a resource that should be involved to improve response by including civil society,^36,49,51^ and the WHO recommends that community health workers and other actors within society should be utilized to help raise the awareness in the population about how to manage shocks.^35^ Both the European Commission and the experts interviewed emphasized that people who work with healthcare need to be given proper support and incentives at all times.^40^ The experts elaborated that support, including satisfactory financial compensation, is important during stable times to ensure that professionals are motivated to work efficiently and is crucial during shocks when fear may cause health workers to leave their stations.

### Information

The continuous collection of data to improve preparedness and response to both long-term changes and shock was a recurring characteristic.^4,19,27,30,33^ Five articles discussed the need for good quality surveillance data so that policies focus on the right areas and make the right decisions to predict changes and shocks that are likely to affect the health system.^20,27,28,40,43^ Information was also mentioned as fundamental to how quickly a health system can adapt to a change.^20^ The need to analyse previous experiences to guide future response was also raised^4,19,20,32,42^ and could include learning or cooperation with other sectors or countries who have experienced similar shocks in order to guide plans for improved response.^19,21,34^

### Leadership and Governance

The building block of leadership and governance was mentioned by 20 sources. Accountability and transparency^21,40,43^ together with equity^43,44^ were emphasized as important responsibilities. According to the expert consultations, equity was highlighted as an aspect that is often lost during times of a shock. One expert stated that new strategies that are made hastily in the need for a quick fix often miss equity aspects. Six articles stated that a system needs the capacity to predict future challenges to effectively respond and to adjust the system when needed.^20,21,27,34,40,45^ This capacity should be built during stable times and not during shocks when the health system is strained and needs to focus on response.^4,8,21,27,32,38^ Three articles underscored that new strategies should be developed based on previous experiences and lessons learned should be efficiently implemented.^27,35,45^ Multiple articles stated that the right actors, both within and outside the health sector and at the international level, should be identified to tackle context-specific challenges^19,27,35,37,42^; the engagement of the right authorities and the population was seen as essential to build health system resilience, for instance, in the implementation of strategies and the response.^19,27,35,37,41,42^ Designing institutions to govern for resilience, for example by making health system governance polycentric, was mentioned as a way to spread the impact of a shock and in that way foster resilience.^20,46^ Local governance and strong community engagement were highlighted as an investment to anticipate uncertain shocks.^46^

### Medical Products, Vaccines, and Technologies

The characteristics related to medical products, vaccines and technologies were mentioned in 2 documents and by one expert.^27,33^ They mentioned that accessibility to medical products is critical for a health system to function well, yet essential medicines remain unaffordable or inaccessible in lower income countries, according to the WHO.^47^ The emphasized challenge was that some essential medical products are not attractive to produce due to small monetary rewards for pharmaceutical companies, and their research and development is thus underfunded.^27,33^ An expert indicated that the incentives for the production of medicines either should be changed or monetary compensation should be increased to incentivize pharmaceutical companies to produce essential medicines. All 3 sources called for a more sustainable production of medical products and technological solutions with improved incentives.^27,33^

### Service Delivery

Nine articles stated the importance of provision of additional services to the entire population during a shock while maintaining everyday services.^4,8,20,21,33-35,38,46^ Focusing on preventive efforts, such as public health interventions, during stable times was identified as one strategy for a health system to be well prepared for shocks and changes, although most shocks were seen as difficult to predict and prevent.^4,28,33,43^

## Discussion

Our study found that in the literature and among health system experts, health system resilience is described mainly in relation to adaptation, maintenance, absorption, learning, transformation, withstanding and responding to shocks. The goals and functions of the health system were reflected in the characteristics of resilience,^1^ and the importance of good governance, effective and reliable information systems, and a resilient workforce were emphasized. Although sudden shocks were identified as the main driver of resilience, adjusting to long-term changes in the health system was also stressed as an important characteristic of resilience that was tied closely to the WHO building block of leadership and governance.

Out of all the full text articles read during the screening, no single description of resilience was referred to consistently. The most common description referenced was the description by Kruk et al – “*The capacity of health actors, institutions, and populations to prepare for and effectively respond to crises; maintain core functions when a crisis hits; and, informed by lessons learned during the crisis, reorganise if conditions require it”*^4^ – yet several authors^37,48-51^ who refer to Kruk et al did not include the third element of the description on learning. The other descriptions, some of which came after the description by Kruk et al and may have thus been influenced by it, all lack this component. Although it could be argued that the element of learning is not as important as the other elements, the opposite was observed when analysing the characteristics of health system resilience and including the perspectives from the expert consultations. Learning was often discussed within the *Information* and *Leadership and governance* building blocks and was mentioned by all experts as an important element, allowing the health system to evolve by learning from previous experiences, thereby increasing the system’s strength and guiding policy-making.

The focus on leadership and governance in both the published literature and by experts is not surprising, given that governance is concerned with how a health system and its actors function and perform. Health systems are dynamic systems that are influenced by the context, the values and principles that the system is built upon, and the variety of interactions among actors and the system.^45,52^ The capacity and breadth for a health system to learn and evolve will depend to a great extent on the decisions that formal and informal actors in the health system are able or willing to take, in any given context. This was apparent in the recurring theme in the results of cooperation with and dependence on other sectors and actors outside of the health system. Effective and responsible coordination of operations working towards a common goal, within the building blocks and with sectors outside the health system, points to the influence of decision-makers and actors in creating change in the system.

The remaining 5 building blocks were discussed with similar frequency with a notable exception of the building block of *Medical products, vaccines and technologies*. Ignoring this building block in the discussion of health system resilience would mean excluding one of the key foundations.^33^ A health system cannot be fully resilient without the availability of essential medicines or without the development of new products. A newly developed, not yet licensed Ebola vaccine was introduced as an emergency strategy to try and stop the 2018 Ebola outbreak in the Democratic Republic of Congo that is still ongoing, and more than 110 000 people have so far been vaccinated.^53^ This is an illustration of how strengthening the provision of medicines like vaccines may help build resilience in a shocked health system. Further, the emergence of antibiotic resistance is a challenge that is closely linked to the building block of *Medical products, vaccines and technology.* It is a slowly emerging disaster that is predicted to become one of the largest health challenges if health systems cannot maintain everyday resilience.^54^ Antibiotic resistance, similar to any other challenge, is also an example of where resilience cannot be tackled in isolation but rather must be addressed as an combination of all the building blocks.

The interactions among the blocks are what make up a resilient health system. The linkages among the blocks, including the role of people as actors who drive the system forward, are essential to realize a resilient health system.^36^ These linkages can be noted in the characteristics that are identified under *Service delivery*, which were often not specific to health system resilience but to health system strengthening in general. Service delivery is the main output of a health system and is dependent on the functions of the other 5 building blocks.^36^ If the other 5 building blocks do not function, service delivery fails, and resilience has not been reached. Health systems are dependent on the interactions among the building blocks to maintain service delivery during everyday challenges and shocks.

It has been argued that the concept of health system resilience is limited to context-specific situations, and, therefore, a single description may be problematic.^55,56^ The diversity of the challenges and shocks and the degree of vulnerability of health systems make it difficult to understand and apply universal characteristics to specific health systems. General guidance for what health system resilience entails and its general characteristics could help a health system to prioritize its actions and strategies within its own context and reach its own resilience goal, thereby contributing to resilience globally. This requires that the concept of resilience is flexible, to effectively adapt to everyday stressors and shocks.^55^

The adoption of health system resilience as a construct has led to criticism about whether it can be applied to complex adaptive systems,^57^ and concern that it is no more than a catchphrase.^17,55^ There has been criticism for seeing resilience as a linear process, where health systems are expected to return to an original state, rather than accommodate change.^16^ It is worth noting that many of the keywords and descriptions from the literature included in this study focused on coping and bouncing back from shocks, rather than learning or system transformation. The implication is that there is a ‘normal’ state that health systems can—and should—return to, and disregards existing challenges and deficiencies of the system that could return it to a continually vulnerable state. This can be further amplified if prescriptive adaptation strategies are suggested for resilience without taking the context of the health systems values, principles, or goals into consideration.^58^ Yet the frequent use of the keyword *adapt,* and the inclusion of the words *transform* and *learn* could indicate a growing emphasis on thinking about health systems as continuously adapting to shocks and stresses, leading to a richer understanding of how to create resilience within health systems.^57^

The ambiguity in terminology in the literature and the absence of a common understanding of health system resilience is comparable to the criticism around the introduction of the term sustainability in development in the 1990s.^21^ Sustainability was criticised for being a catchphrase^59^ and lacking consistency in its interpretation and clarity, leading to concerns about its political usefulness.^60^ Yet the shift in the use of the term from an environmental focus to a human focus, coupled with a growing concern about the ecology of the planet, brought sustainability to the mainstream,^61^ and 2 decades later, sustainability is used in multiple disciplines and is the central image of the United Nations Sustainable Development Goals of the Agenda 2030.^59,62^ To further strengthen the concept of health system resilience, experiences from other disciplines could be helpful to ultimately find a common way to describe and apply the concept in literature. It remains to be seen if health system resilience will be adopted as a useful concept that shapes the way that actors perceive and design health systems in the future.

## Limitations

Two broad search terms were used to identify a greater number of articles during the search. Relevant articles that did not explicitly mention resilience or health systems or articles on resilience that were not published in English may have been missed. Our own interpretation and subjectivity of choosing the keywords and characterizing resilience within the WHO 6 building block framework may have caused a limitation of reproducibility. The building block framework was chosen as it is a commonly used framework, although it places equal emphasis on all building blocks and does not take into account the context of the health system. Choosing another framework to use for the analysis may have shifted the focus towards different dimensions of health systems. In addition, the experts selected for consultation may not be generally representative of health system resilience practitioners, and therefore reflect only some of the ideas regarding health system resilience.

## Conclusion

Health system resilience is an emerging concept within health system research, and descriptions of health systems resilience vary in the existing literature. A review of the literature suggests that long-term changes, learning from previous experiences, and everyday resilience are considered important aspects of resilience. Additional case studies of health system successes and failures in diverging contexts may be a starting point to create a shared understanding of the concept of resilience and to clarify its meaning.

## Acknowledgements

We give special thanks to the 4 experts who participated in the consultations for their valuable contribution. In addition, we thank Helle Mölsted-Alvesson for her methodological input on the expert consultations.

## Ethical issues

Ethics approval was not sought for the study since the literature search used published data, the expert consultations collected no personal information and no sensitive topics were discussed. Participation was voluntary. All participants gave oral informed consent to participate in the study and were informed of their right to withdraw at any time during the consultation. No personal data were stored.

## Competing interests

Authors declare that they have no competing interests.

## Authors’ contributions

MF, SE, JvS, and DDS conceptualized and designed the study. MF and SE collected and analyzed the data. MF, SE, JvS, and DDS wrote the manuscript. All authors agreed to the final draft of the manuscript.
